# Importance of Fluorine in High Voltage Electrolytes for LNMO||SiGr Cell Chemistry

**DOI:** 10.1002/smll.202505254

**Published:** 2025-07-10

**Authors:** Maike Leopold, Felix Pfeiffer, Elisabeth Christine Muschiol, Christian Wölke, Peng Yan, Kai Brüning, Sascha Nowak, Melanie Esselen, Martin Winter, Isidora Cekic‐Laskovic

**Affiliations:** ^1^ Helmholtz‐Institute Münster (IMD‐4) Forschungszentrum Jülich GmbH Corrensstraße 48 48149 Münster Germany; ^2^ Institute of Food Chemistry University of Münster Corrensstraße 45 48149 Münster Germany; ^3^ MEET Battery Research Center University of Münster Corrensstraße 46 48149 Münster Germany

**Keywords:** high voltage electrolyte, lithium ion battery, lithium nickel manganese oxide electrode, non‐flammable electrolyte, silicon graphite electrode

## Abstract

Lithium nickel manganese oxide (LNMO) and silicon/graphite (SiGr) are promising active materials for high voltage lithium ion batteries attributed to the high operating potential versus Li|Li^+^ of LNMO and the high specific discharge capacity of silicon. However, this cell chemistry exhibits rapid capacity fading, primarily attributed to electrolyte decomposition at the high operating voltage of 4.9 V. Here, a fluorinated electrolyte containing lithium hexafluorophosphate as conducting salt, as well as fluoroethylene carbonate and methyl (2,2,2‐trifluoroethyl) carbonate as electrolyte solvents is introduced. The influence of the selected solvents on the interphase formation and galvanostatic cycling performance is analyzed using complementary electrochemical, spectroscopic, and safety‐related techniques. The presence of fluorinated solvents enables a high oxidative stability of an electrolyte up to 5.0 V versus Li|Li^+^ and effective interphase formation. In comparison to cells with non‐fluorinated electrolytes, the galvanostatic cycling performance demonstrates a considerable improvement, leading to a doubling of the achievable cycle life. Roll‐over failure observed in the electrolyte with non‐fluorinated solvents could be effectively suppressed for over 300 cycles and the resulting electrolyte formulation with fluorinated solvents is non‐flammable. Additionally, by fine‐tuning the electrolyte formulation, the extent of acetylcholinesterase inhibition, an indication of substance toxicity of the aged electrolyte could be reduced.

## Graphical abstract

A non‐flammable fluorinated electrolyte is developed for a high voltage LNMO||SiGr (20 wt.% Si) cell chemistry to improve the cycle life until 80% SOH by 56% compared to the reference cell chemistry. This electrolyte prevents the roll‐over effect and suppresses side reactions, resulting in an effective solid electrolyte interphase (SEI) and cathode electrolyte interphase (CEI) formation.

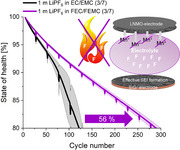

## 1 Introduction

The rapidly growing demand for lithium ion batteries (LIBs) for electric vehicles presents an incredible opportunity to push the boundaries of technology and innovation, as these batteries are being developed to deliver unmatched specific energy, energy density, and production cost efficiency, marking a new era of sustainable energy advancements.^[^ ^1^, ^2^ ^]^ On the cathode side, the high voltage lithium nickel manganese oxide (LiNi_0.5_Mn_1.5_O_4_) is a promising candidate to achieve these requirements. The advantages of LNMO electrodes include a reversible specific capacity of 147 mAh g^−1^ and a higher theoretical energy density of ≈650 Wh kg^−1^, which is 1.31 times higher than that of LiFePO_4_ electrode (495 Wh kg^−1^) due to the high discharge voltage plateau of 4.7 V.^[^ ^1^, ^3^, ^4^, ^5^, ^6^ ^]^ Also important in terms of cost volatility and environmental impact is the complete exclusion of Co as a critical raw material.^[^ ^1^, ^6^ ^]^ However, the use of LNMO‐based cells leads to substantial capacity fading due to the electrolyte decomposition and dissolution behavior of Mn and Ni.^[^ ^3^, ^4^, ^7^ ^]^ To achieve a higher energy density also on the anode side, silicon was added to the graphite material.^[^ ^2^, ^8^ ^]^ Silicon has a high theoretical specific capacity of 3590 mAh g^−1^ at room temperature for the Li_3.75_Si phase, which is approximately a tenfold increase in specific capacity compared to state‐of‐the‐art graphite with a specific capacity of 372 mAh g^−1^.^[^ ^2^, ^9^, ^10^ ^]^ Furthermore, silicon is characterized by its environmental friendliness, low cost, and high natural abundance.^[^ ^2^, ^9^, ^10^ ^]^ However, there are also several challenges such as a high volume change of up to 300% during (de‐)lithiation, which is roughly thirty times higher than the volume change of graphite and can lead to particle cracking.^[^ ^2^, ^9^, ^10^ ^]^ Particle cracking results in capacity loss, an increase in internal resistance, lower Coulombic efficiency (CE), and solid electrolyte interphase (SEI) instability.^[^ ^2^, ^9^, ^10^ ^]^ Due to the disadvantages of silicon, it is combined with graphite to leverage the advantages of both materials, especially in terms of volume expansion and theoretical capacity.^[^ ^11^ ^]^

One way to improve the performance and safety of the LNMO||SiGr cells is to optimize the electrolyte. Fluorinated solvents have drawn considerable attention due to their unique physiochemical properties including low viscosity, low‐temperature adaptability, and wide electrochemical stability window.^[^ ^12^, ^13^, ^14^ ^]^ In addition, the introduction of a trifluoromethyl group (CF_3_), a strong electron‐withdrawing group, into a molecule can considerably lower the highest occupied molecular orbital (HOMO) and lowest unoccupied molecular orbital (LUMO) energies, thereby improving the oxidative stability of the electrolyte at high voltage.^[^ ^13^, ^15^, ^16^, ^17^ ^]^ This ensures high voltage interfacial/interphasial stability by forming a fluorine‐rich SEI and also improved electrochemical stability on the cathode side.^[^ ^13^, ^16^ ^]^ In addition, there are drawbacks to fluorinated solvents when it comes to per‐ and polyfluoroalkyl substances (PFAS). PFAS are a class of organic compounds characterized by carbon‐fluorine bonds, either fully (perfluorinated) or partially (polyfluorinated) replacing hydrogen atoms.^[^ ^18^, ^19^ ^]^ Concerns about environmental aspects and toxicity of fluorinated organic compounds have triggered a re‐evaluation of PFAS applications.^[^ ^18^, ^20^ ^]^ The recent EU regulation on PFAS has garnered considerable attention within the LIB research community, particularly regarding the restrictions on the use of PFAS‐containing components.^[^ ^18^, ^21^, ^22^ ^]^

Due to the unique properties of fluorinated solvents, fluoroethylene carbonate (FEC) and methyl (2,2,2‐trifluoroethyl) carbonate (FEMC) were selected as electrolyte solvent/co‐solvent for a LNMO||SiGr cell chemistry. FEC is a well‐known electrolyte additive/co‐solvent that enhances the anodic stability and film‐forming ability on the anode surface compared with ethylene carbonate (EC).^[^ ^11^ ^]^ The improved performance is attributed to the higher oxidative stability of FEC, which results in the formation of a more effective SEI.^[^ ^23^, ^24^, ^25^, ^26^ ^]^ Similarly, FEMC was selected due to its high oxidation stability compared to ethyl methyl carbonate (EMC), attributed to its lower HOMO energy level, which enhances electrolyte compatibility with high voltage cathodes.^[^ ^26^, ^27^, ^28^ ^]^ Furthermore, the reduction reaction of FEMC, facilitated by the CF_3_ functional group, enables the formation of fluorine‐rich organic compounds in the SEI.^[^ ^28^ ^]^ Incorporating fluorine into the electrolyte promotes the development of an effective SEI, leading to improved electrochemical stability and galvanostatic cycling performance in high voltage cells.^[^ ^28^, ^29^ ^]^

The effect of the combination of FEC and FEMC on the performance of high voltage LNMO||SiGr cells was investigated, comparing them with non‐fluorinated and partially fluorinated counterparts. By employing complementary electrochemical, spectroscopic, and safety‐related techniques, it was demonstrated that the presence of fluorinated electrolytes in considered cell chemistry results in a considerable improvement in galvanostatic cycling performance. In addition, this electrolyte formulation is non‐flammable in nature.

## 2 Results and Discussion

**Figure 1:**
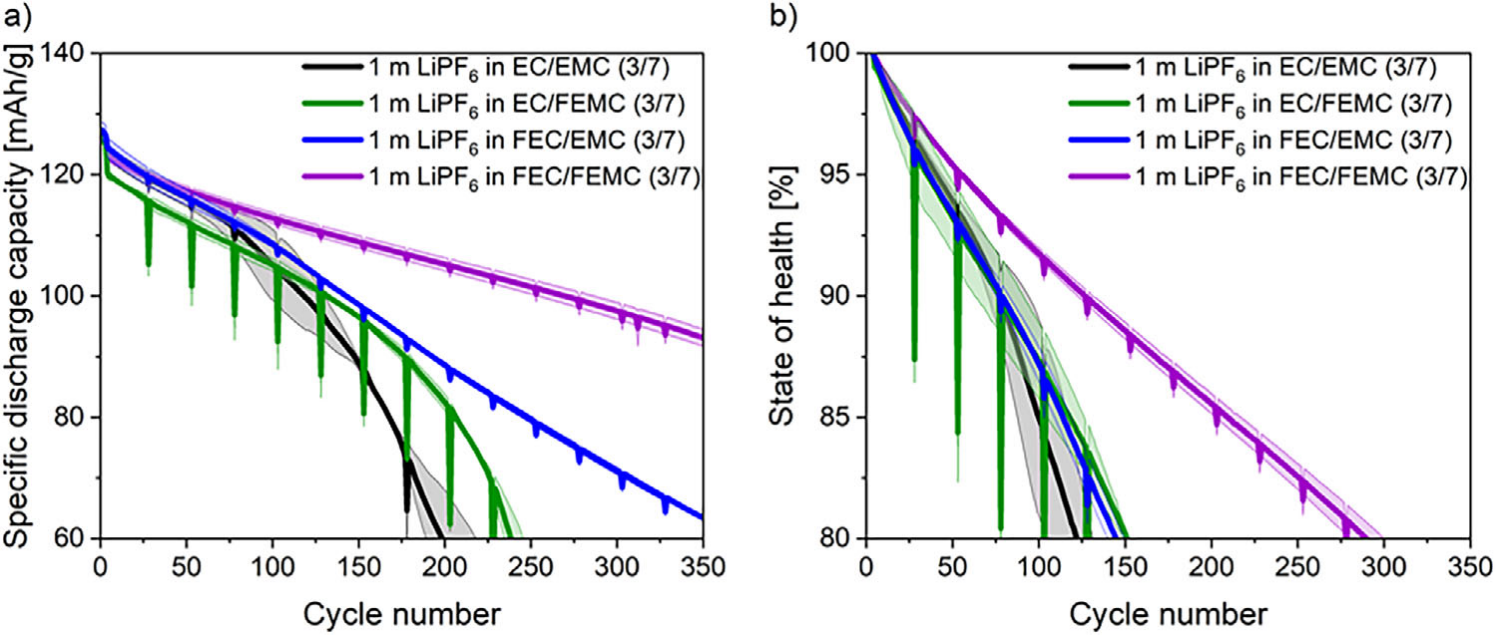
Mean specific discharge capacity versus cycle number a) and state of health versus cycle number profiles b) of LNMO||SiGr (20 wt.% Si) cells with four considered electrolyte formulations.

The poor electrochemical performance of LNMO electrode‐based cells in combination with organic carbonate‐based electrolytes is well known.^[^ ^26^ ^]^ Therefore, this study explores the use of fluorinated carbonate‐based solvents to improve the performance of LNMO‐based LIB cells. To analyze the SEI and cathode electrolyte interphase (CEI), *post mortem* analysis was carried out after 3 and 50 cycles to address the differences in performance between the different electrolyte systems. **Figure** **1** shows the specific discharge capacity a) and state of health (SOH) b) during the first 350 cycles for cells containing four different electrolyte formulations. A cell with the non‐fluorinated reference electrolyte of 1 m LiPF_6_ in EC/EMC (3/7) (named “non‐fluorinated electrolyte”) exhibited a consistent capacity decline during the first 50 cycles. Subsequently, a roll‐over failure was observed, characterized by rapid and abrupt capacity fading.^[^ ^30^ ^]^ The cell achieved only 125 cycles until 80% SOH, due to the poor compatibility of the fluorine‐free organic carbonate‐based solvents with the high voltage cell chemistry.^[^ ^26^ ^]^ To enhance performance, EMC was replaced with FEMC (1 m LiPF_6_ in EC/FEMC (3/7)) due to its good stability with high voltage cells.^[^ ^26^, ^27^, ^28^ ^]^ The substitution resulted in a lower initial capacity and the roll‐over effect could be postponed for 80 cycles. However, the cell still only achieved 150 cycles until 80% SOH, probably due to the notable capacity drop during the 1C steps. Replacing EC with FEC (1 m LiPF_6_ in FEC/EMC (3/7)) in the electrolyte formulation was motivated by the known film‐forming ability of the SiGr electrode.^[^ ^23^, ^24^, ^25^, ^26^ ^]^ This substitution enabled the achievement of the same initial capacity compared to the non‐fluorinated counterpart electrolyte while effectively preventing the rollover effect, resulting in a consistent capacity decline and improved high‐rate performance. However, again only 150 cycles could be achieved until 80% SOH. This indicates that fluorination of only one of the organic carbonate‐based solvents is insufficient to achieve a considerable improvement in the galvanostatic cycling performance of the resulting cell chemistry. By introducing both fluorinated solvents, FEMC and FEC (1 m LiPF_6_ in FEC/FEMC (3/7)) (named as “fluorinated electrolyte”), 290 cycles could be achieved until 80% SOH. The presence of both fluorinated solvents also effectively mitigates the rollover effect during the first 350 cycles. Furthermore, as shown in Figure S1 (Supporting Information), the Coulombic efficiency (CE) of the cells with the fluorinated electrolyte was consistently higher than that of the cells with the non‐fluorinated electrolyte. The CE of the cells with the fluorinated electrolyte exhibited an initial increase over the first 60 cycles before stabilizing. In contrast, the CE of the cells with the non‐fluorinated electrolyte showed a similar trend during the first 60 cycles, but subsequently dropped sharply, aligning with the observed capacity fading from the galvanostatic cycling data. To investigate the extent of transition metal (TM) deposition on the anode, laser‐ablation inductively coupled plasma mass spectroscopy (LA‐ICP‐MS) elemental mapping analysis of harvested SiGr anodes was performed. An increased TM dissolution from the LNMO electrode and subsequent deposition onto the SiGr electrode was observed when using the non‐fluorinated electrolyte compared to the fluorinated counterpart after 50 cycles, as shown in Figure S2 (Supporting Information). This is a further indication of the reason for the fast capacity fading of the cells containing non‐fluorinated electrolytes.

**Figure 2:**
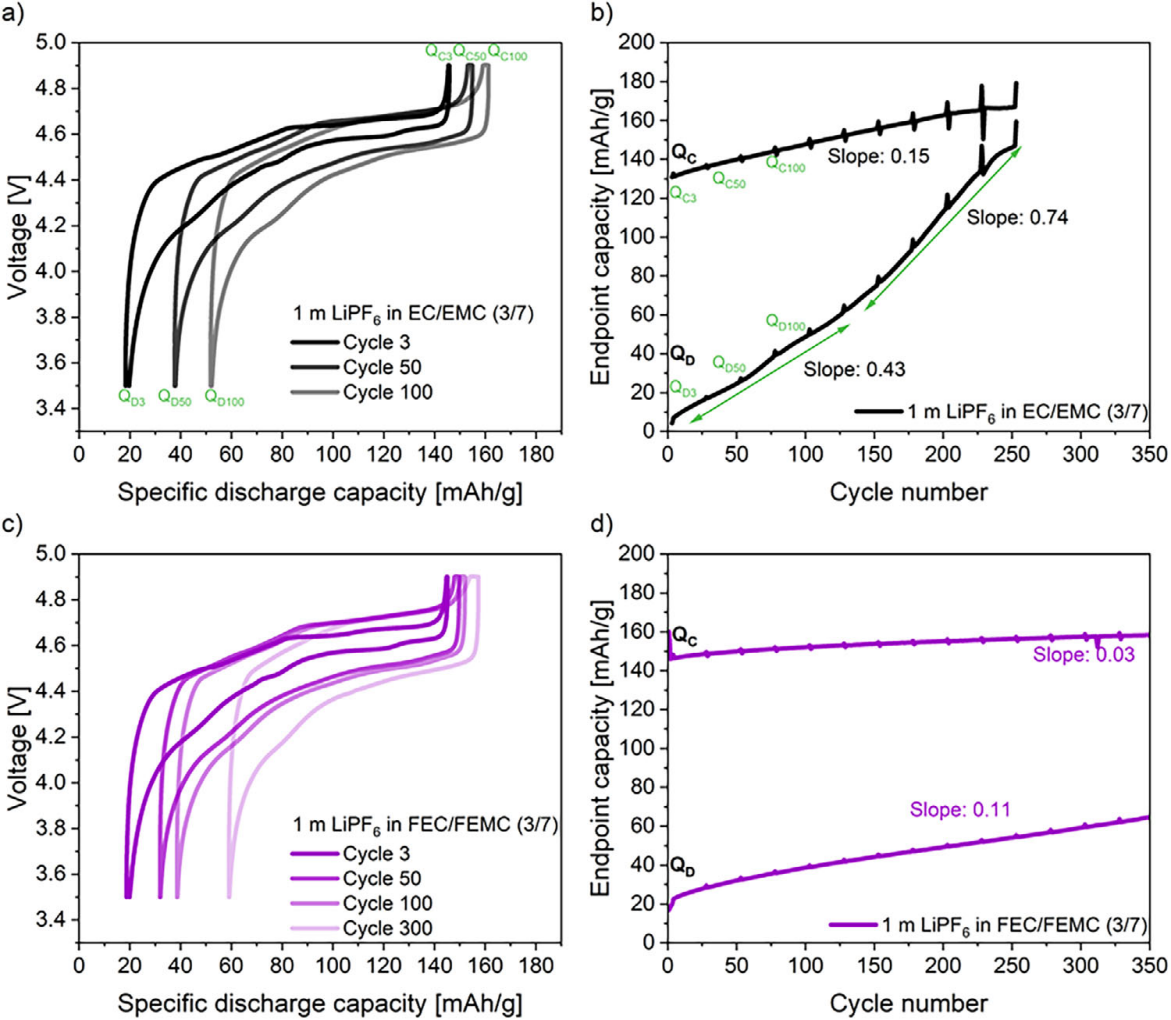
Electrochemical charge–discharge profile of LNMO||SiGr (20 wt.% Si) cells with non‐fluorinated a) and fluorinated electrolyte c) versus specific discharge capacity. The charge and discharge endpoints of LNMO||SiGr (20 wt.% Si) cells with non‐fluorinated b) and fluorinated electrolyte d) are plotted using the cumulative capacity as a function of cycle number.

The same trend of the irreversible capacity drop, as see in the galvanostatic cycling data, can also be observed with the charge–discharge profiles after 3, 50, 100, and 300 cycles of cells containing the non‐fluorinated electrolyte (a) and the fluorinated electrolyte (c) as shown in **Figure** **2**. Moreover, a lower overvoltage was observed with the non‐fluorinated electrolyte compared to the fluorinated counterpart. Subsequently, the capacity marching rate, which indicates the shifts of charge and discharge capacity endpoints as a result of side reactions, was analyzed for the non‐fluorinated and fluorinated electrolytes.^[^ ^31^, ^32^ ^]^ The capacity endpoint of charge (Q_C_) and the capacity endpoint of discharge (Q_D_) were determined based on the literature.^[^ ^31^, ^32^ ^]^ The capacity endpoints shift due to side reactions, with the slope value reflecting the rate of degradation.^[^ ^32^ ^]^ Figure 2 shows the calculated Q_C_ and Q_D_ of the non‐fluorinated electrolyte (b) and fluorinated electrolyte (d). For cells with the non‐fluorinated electrolyte, Q_D_ has a marching rate of 0.43 mAh g^−1^ during the first 125 cycles probably due to the formation of ineffective SEI, which cannot withstand the volume changes of SiGr electrode and leads to the continuous electrolyte decomposition with high Li consumption. The observed drop in CE after 50 cycles further confirms the presence of side reactions at the negative electrode, as shown in Figure S1 (Supporting Information) and the observed detachment of the anode material from the current collector signifies the occurrence of irreversible processes at the anode as shown in Figure S3 (Supporting Information).^[^ ^31^, ^32^ ^]^ In contrast, no loss of coating material was observed with the fluorinated electrolyte during the first 50 cycles as shown in Figure S3 (Supporting Information). After 125 cycles a knee point can be observed and the marching increases drastically to 0.74 mAh g^−1^. The sharp drop in capacity suggests the breakdown of the SEI.^[^ ^32^ ^]^ In addition, a marching rate of 0.15 mAh g^−1^ is observed for Q_C_, indicating an ineffective CEI formation due to parasitic oxidation reactions taking place.^[^ ^31^, ^32^ ^]^

In contrast, cells with a fluorinated electrolyte exhibited considerably improved electrochemical stability. A small increase of Q_D_ with a marching rate of 0.11 mAh g^−1^ and Q_C_ with a marching rate of 0.03 mAh g^−1^ was observed. The small increase in Q_D_ suggests minimal side reactions indicating the formation of an effective SEI layer.^[^ ^32^ ^]^ This nearly constant drop in Q_C_ endpoints with a marching rate of 0.03 mAh g^−1^, attributed to suppressed side reactions, suggests the formation of an effective CEI layer.^[^ ^32^ ^]^ This behavior is characteristic of classical ageing, where Q_D_ endpoints converge very slowly to Q_C_ endpoints. In summary, the fluorinated electrolyte facilitated the formation of robust SEI and CEI layers, which resulted in reduced electrolyte decomposition, minimized lithium immobilization, and suppressed parasitic reactions at the cathode, leading to enhanced overall cell performance.^[^ ^31^, ^32^ ^]^

**Figure 3:**
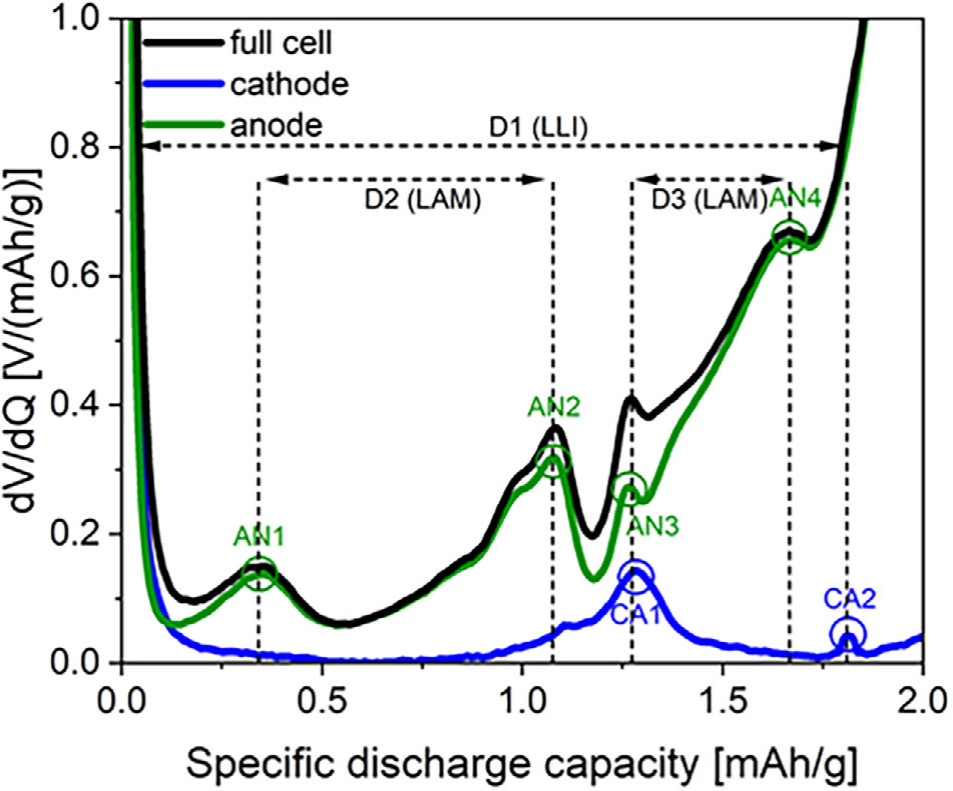
Differential voltage versus capacity curves during the first discharge in a three‐electrode LNMO||SiGr (20 wt.% Si) cell with non‐fluorinated electrolyte.

**Figure 4:**
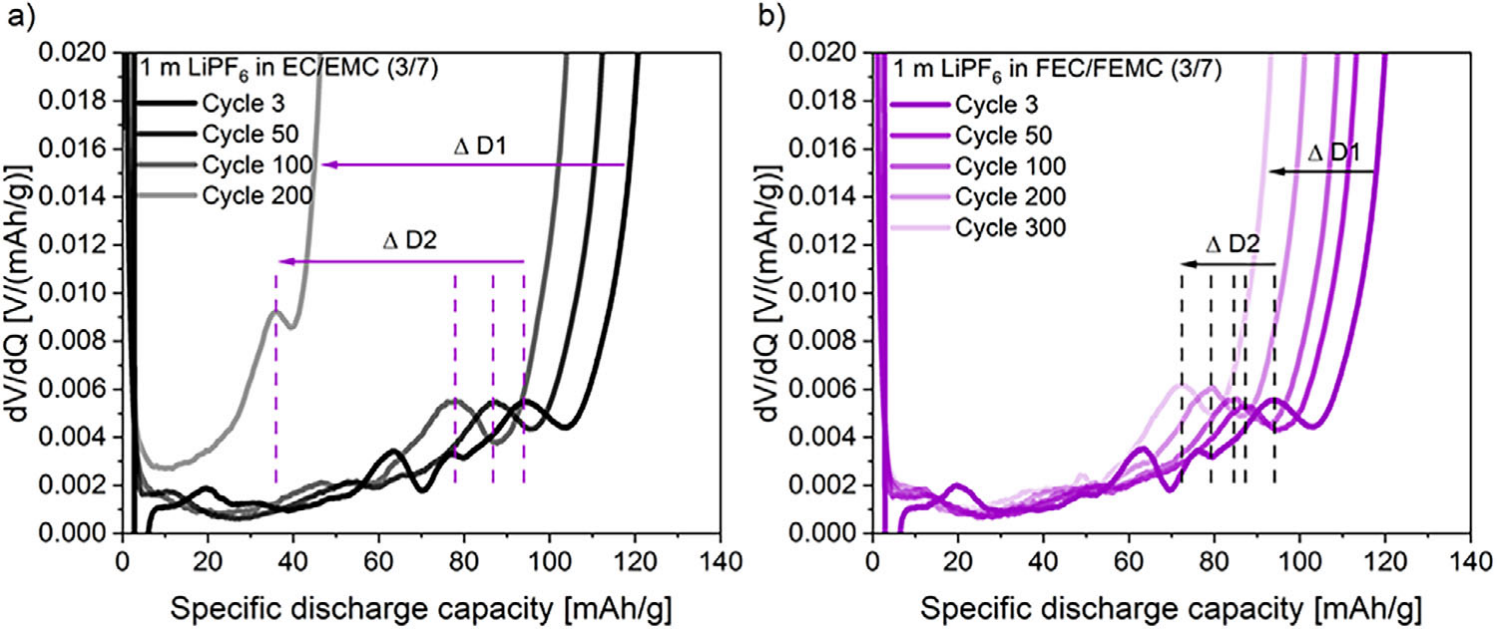
Differential voltage versus discharge capacity curves of LNMO||SiGr (20 wt.% Si) cells with non‐fluorinated (a)and fluorinated solvents (b).

Derivate voltage analysis (DVA) was performed to elucidate the contributions of the individual LNMO and SiGr electrodes to capacity fading during galvanostatic cycling. Utilizing a three‐electrode cell setup, DVA enabled the differentiation of individual potential contributions from the positive and negative electrode to the cell voltage^[^ ^33^ ^]^ and estimated the loss of lithium inventory (LLI) and the loss of active material (LAM) during ageing.^[^ ^31^ ^]^ **Figure** **3** shows the DVA profile of a three‐electrode cell setup to distinguish between the contributions of the SiGr electrode (AN) and the LNMO electrode (CA) to different peaks. The DVA for the anode exhibits four distinct peaks: AN1 – 4, while the cathode shows two peaks CA1 and CA2. Notably, discernible distances exist between these peaks. These peak separations are proportional to the active material capacity. Specifically, the distance D1 can be used to estimate the LLI. The loss of the anode active material can be estimated by the distance D3 between AN3 and AN4.^[^ ^31^, ^32^ ^]^ However, no discernible peak shifting of CA1 and CA2 was observed, due to the overlap of the CA1 and AN3 peaks, precluding any definitive conclusions regarding the loss of cathode material. The DVA for the pouch‐cell experiments using the non‐fluorinated electrolyte is shown in **Figure** **4**a. A considerable shift of D1 indicates a high irreversible LLI loss. There is also a strong shift in the other anode peaks, which is shown in D2 as an example, indicating a high loss of the anode material. After 100 and 200 cycles, the peaks are no longer observable, indicating an irreversible anode active material loss, which can also be clearly seen in the dQ/dV versus capacity plot (Figure S4, Supporting Information). For the cells with fluorinated solvents, a substantially smaller shift D1 is observed, which indicates a small irreversible LLI. Also, there is a lower shift in D2, which reflects a smaller anode active material loss.^[^ ^31^, ^32^ ^]^ This further supports the conclusion that in the case of the non‐fluorinated electrolyte, an ineffective SEI is a major cause of the poor electrochemical performance of the resulting cells.

**Figure 5:**
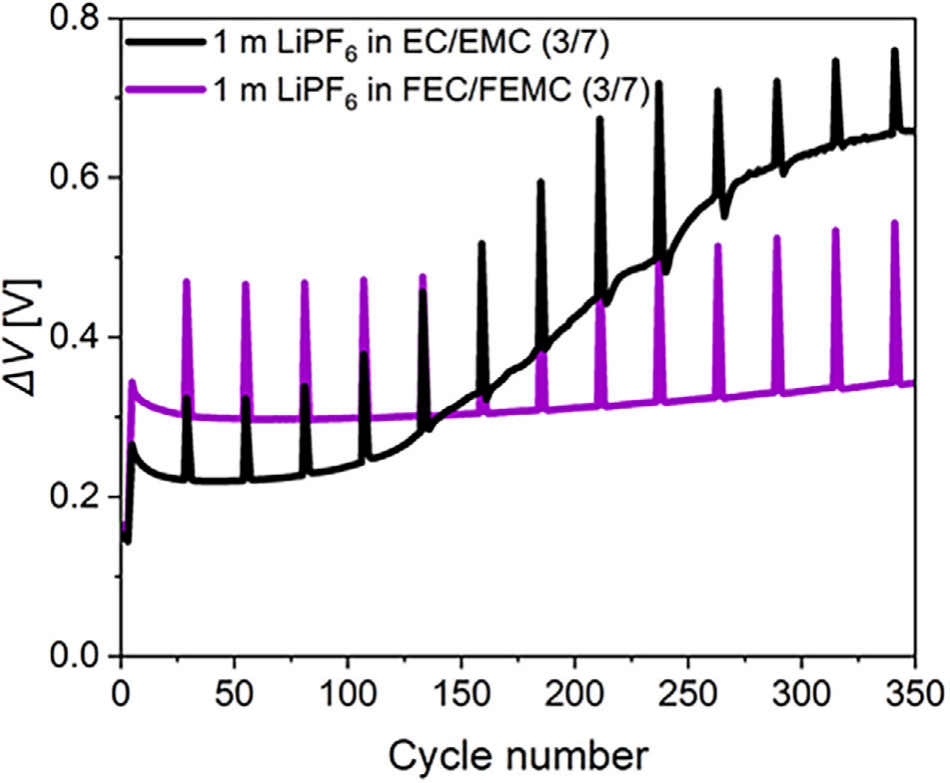
Cell polarization (*∆V*) as a function of cycle number of LNMO||SiGr cells with electrolytes containing non‐fluorinated and fluorinated solvents.

The difference between the average charge and the average discharge voltage (Δ*V*) is plotted against the cycle number for the first 300 cycles and presented in **Figure** **5**. Δ*V* can give indirect information about the impedance of a cell, i.e., higher Δ*V* values imply a higher cell impedance.^[^ ^34^ ^]^ Cells with the non‐fluorinated electrolyte formulation exhibited lower polarization during the initial 130 cycles compared to cells with their fluorinated counterpart. The evolution of the cells with the non‐fluorinated electrolyte shows a lower overvoltage during the first 3 cycles with regard to the low C‐rate of 0.1 C, followed by a decreasing overvoltage during the next 25 cycles. However, after 50 cycles, the trend shifts as the roll‐over effect begins, leading to a steady increase in Δ*V* during galvanostatic cycling. This can be attributed to rising impedance, as evidenced by increased voltage polarization.^[^ ^34^ ^]^ In general, the rise in impedance can result from an ineffective SEI and CEI formation and could be an explanation for the rollover effect after 50 cycles. The cells with the non‐fluorinated electrolyte also showed an improved specific discharge capacity during the first 15 cycles, which matched the lower Δ*V* value. In comparison, the cells with the fluorinated electrolyte have an almost constant Δ*V* upon galvanostatic cycling after the three formation cycles, which is also reflected in the slow decrease of the specific discharge capacity. These findings suggest that the cells with the fluorinated electrolyte facilitate the formation of a more effective interphase layer.^[^ ^35^ ^]^

**Figure 6:**
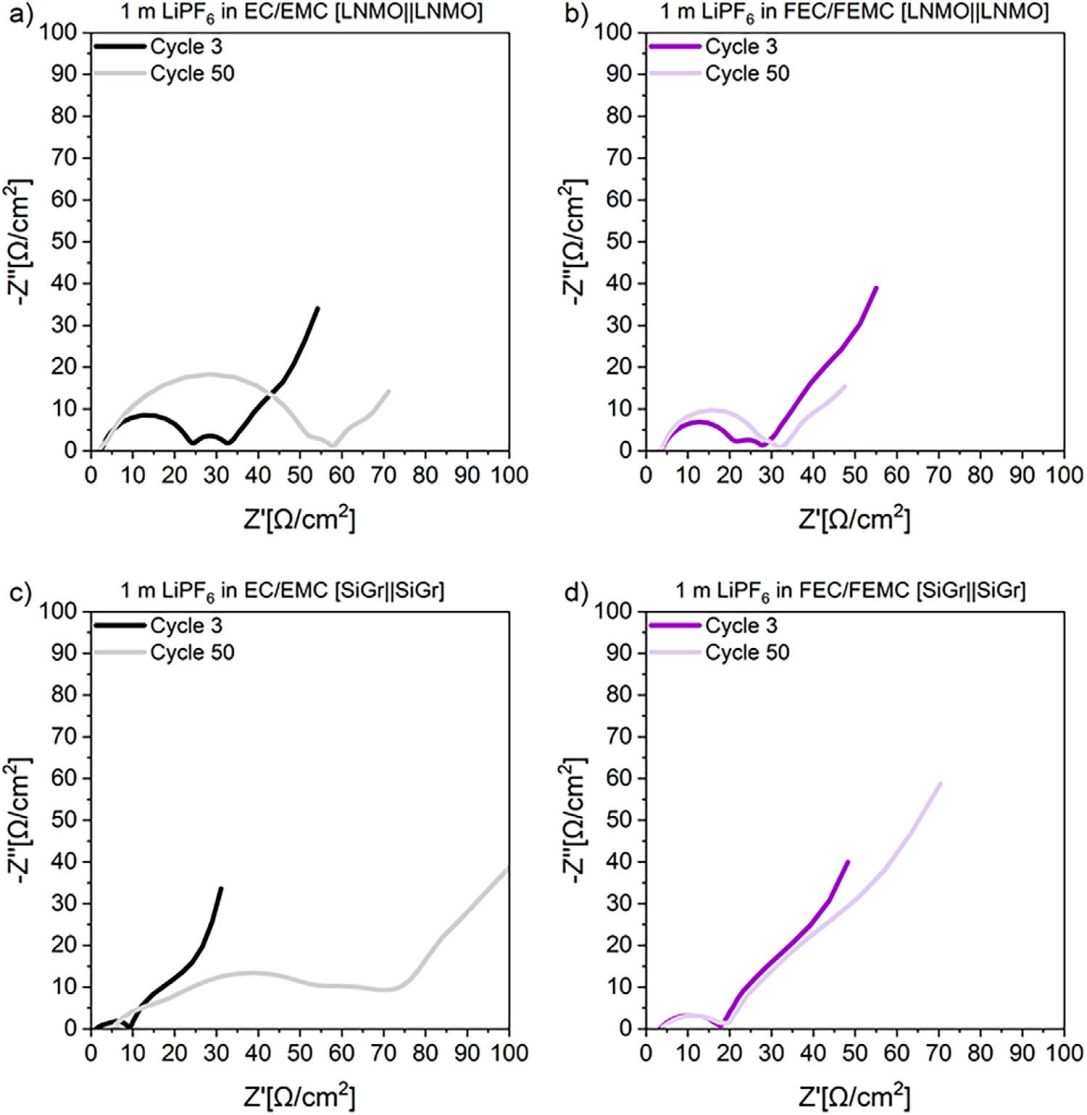
Nyquist plots of reassembled symmetric LNMO||LNMO a,b) and SiGr||SiGr c,d) cells containing non‐fluorinated and fluorinated electrolytes after 3 and 50 cycles.

In order to gain a deeper understanding of the interphase formation of the non‐fluorinated and fluorinated electrolyte, electrochemical impedance spectroscopy (EIS) measurements were conducted after 3 and 50 cycles, as depicted in **Figure** **6**.^[^ ^32^ ^]^ To distinguish between the effects on the cathode and anode, EIS measurements were performed in symmetric cells using the method described by *Petibon* et al.^[^ ^36^ ^]^ Impedance spectra typically feature two semicircles in the high to middle‐frequency region, representing the interfacial resistance associated with surface film (R_f_) formation and charge transfer (R_CT_).^[^ ^32^, ^37^ ^]^ Additionally, a straight line appearing at the low‐frequency region corresponds to the Li^+^ ion diffusion within the bulk.^[^ ^32^, ^37^ ^]^ The following processes can be observed in the symmetric LNMO||LNMO cells. After 3 cycles, there is a lower resistance with the non‐fluorinated electrolyte compared to the fluorinated electrolyte, which is consistent with the lower overvoltage and the higher specific discharge capacity observed with the non‐fluorinated electrolyte. After 50 cycles, the opposite behavior can be observed, as the resistance of the non‐fluorinated electrolyte in comparison to the fluorinated electrolyte increases considerably more, which indicates an instability of the interphase between the electrode and electrolyte due to the continuous decomposition of the electrolyte. This corresponds with the onset of the roll‐over failure after 50 cycles.^[^ ^38^ ^]^ Due to a more effective CEI formation with the fluorinated electrolyte, there is a considerably smaller increase in the R_f_ and R_CT_ up to 50 cycles.^[^ ^37^ ^]^ A similar trend is observed with the symmetric SiGr||SiGr cells. After 3 cycles, the cell with the non‐fluorinated electrolyte shows lower R_f_ and R_CT_ values compared to the cell with the fluorinated electrolyte. However, after 50 cycles, a considerable increase in both R_f_ and R_CT_ values is detected and a third semicircle emerges at a lower frequency. The extent of the third semicircle at the *x*‐axis represents the resistance of the electrode‐to‐current collector interface^[^ ^32^ ^]^ and it is probably caused by the detachment of the anode. This indicates an ineffective SEI. In contrast, the fluorinated electrolyte shows no such increase in resistance, suggesting effective SEI formation during the initial formation cycles.

**Figure 7:**
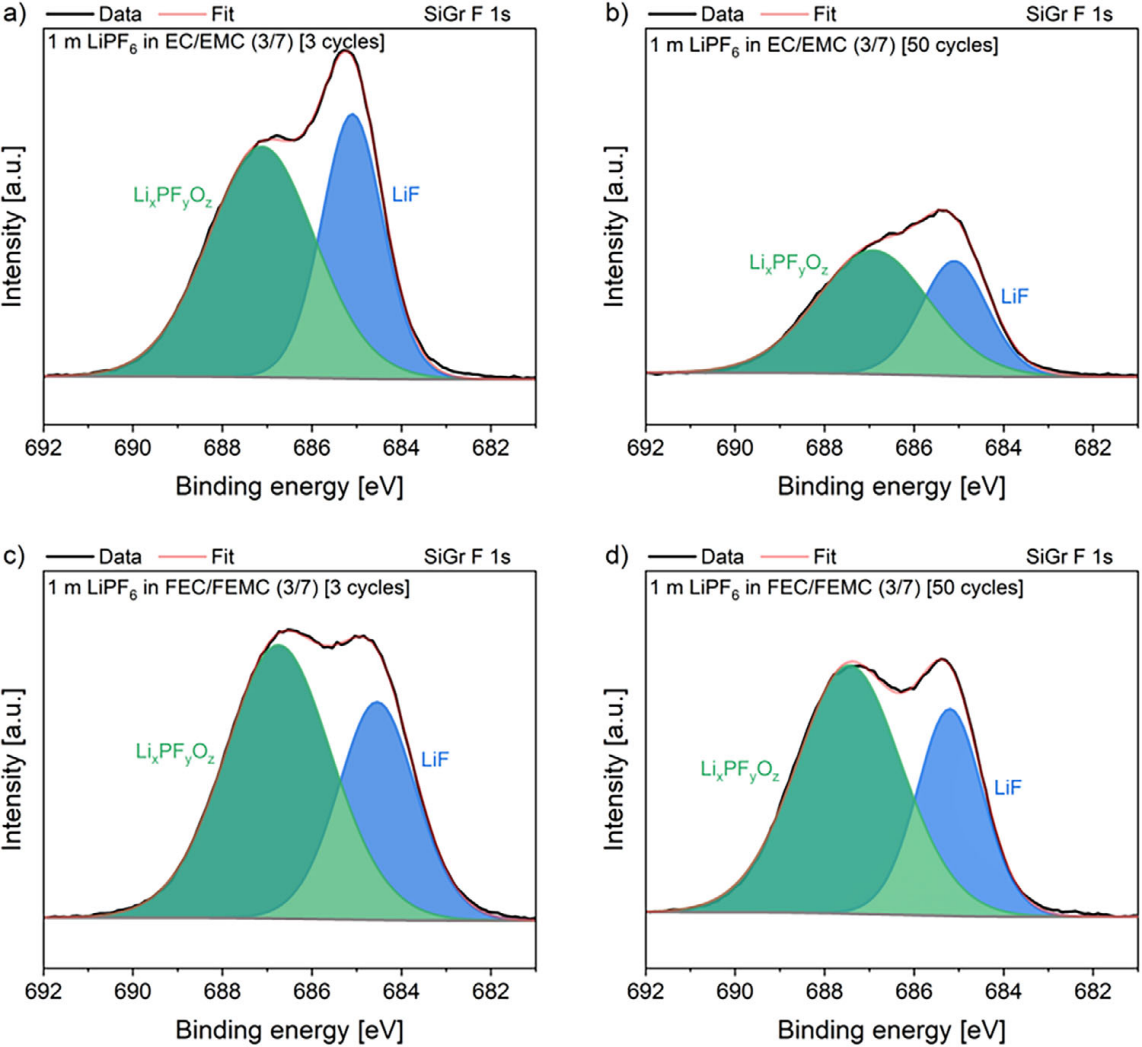
Fitted core F 1s spectra for harvested SiGr electrodes with non‐fluorinated a,b) and fluorinated electrolyte c,d) after 3 and 50 cycles.

**Figure 8:**
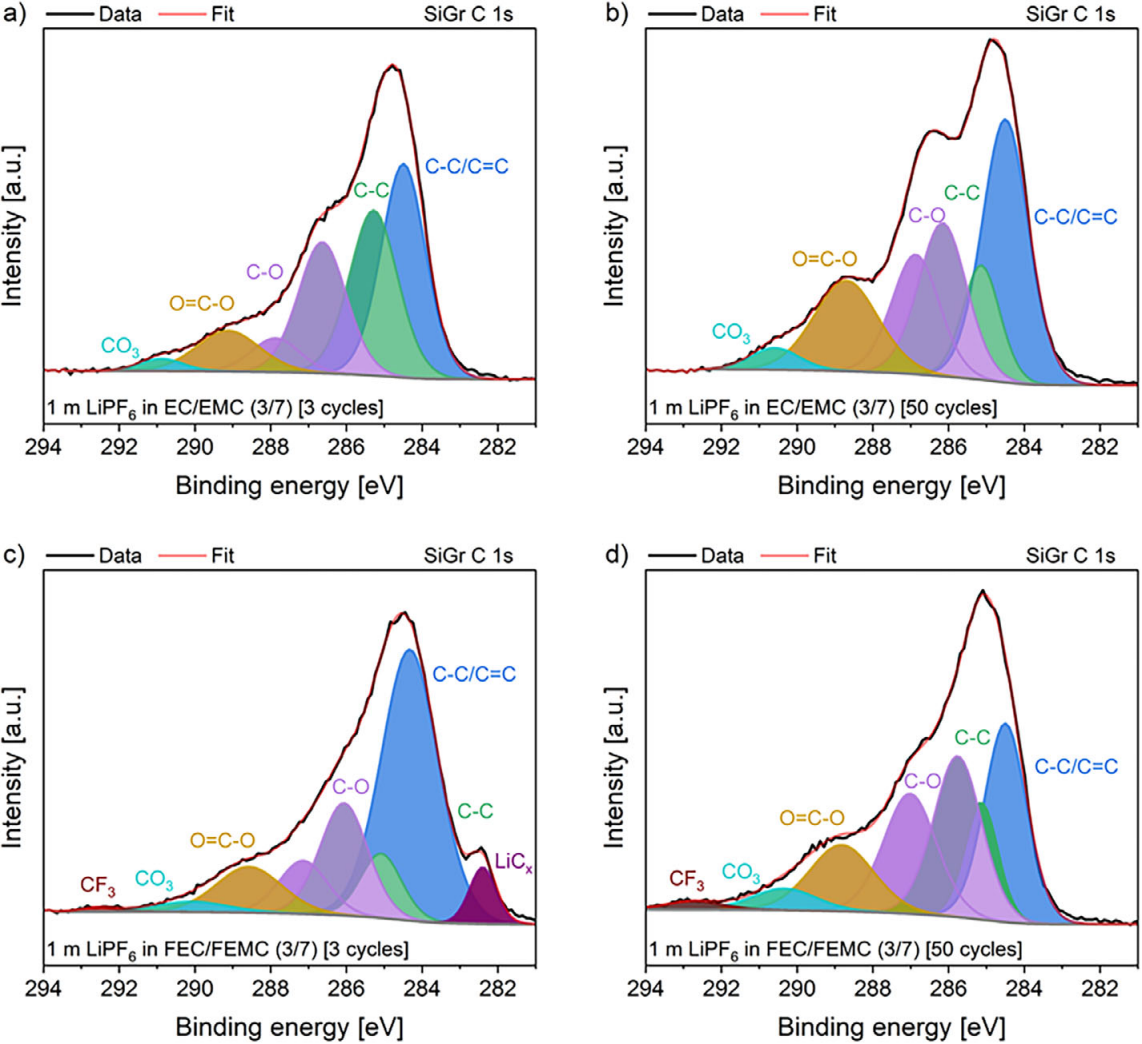
Fitted core C 1s spectra for harvested SiGr electrodes with non‐fluorinated a,b) and fluorinated electrolyte c,d) after 3 and 50 cycles.

X‐ray photoelectron spectroscopy (XPS) analysis was carried out to gain insight into the compositional differences of SEI and CEI formed in the presence of fluorinated and non‐fluorinated electrolytes after 3 and 50 cycles. The F 1s spectra of SiGr electrodes of the cells with the non‐fluorinated and fluorinated electrolyte exhibit two peaks at ≈687.2 and ≈685.1 eV (**Figure** **7**). The peak observed at ≈687.2 eV is correlated to Li_x_PF_y_O_z_ and the peak at ≈685.1 eV to LiF,^[^ ^39^, ^40^ ^]^ which arise from the decomposition of LiPF_6_ and the latter also being a decomposition product of FEC.^[^ ^38^, ^41^ ^]^ Interestingly, a similar intensity amount of the Li_x_PF_y_O_z_ and LiF signals was observed with both electrolytes, despite the high fluorine content of the fluorinated electrolyte, and showed practically no change after 50 cycles. The C 1s spectra of the SiGr electrodes exhibit characteristic peaks from organic carbonate decomposition products like C─C, C─O, C═O, and CO_3_ species (**Figure** **8**).^[^ ^42^ ^]^ In general, there is a clear difference between the cells with the different electrolytes after 3 cycles. The observed substantially higher intensity of the peak which corresponds to the C─C species and a higher relative peak intensity of C─O and O═C─O of the cells with the non‐fluorinated electrolyte compared to the fluorinated electrolyte strongly suggests a greater abundance of organic carbonates in the SEI with the non‐fluorinated electrolyte. In addition, the intensity of the C─C/C═C peaks is notably lower in the case of the non‐fluorinated electrolyte compared to the fluorinated electrolyte and the relative intensity of the O═C─O peaks and CO_3_ peaks is approximately the same. Furthermore, there is a small CF_3_ peak observable in the case of the fluorinated electrolyte, which is correlated to the use of FEMC as an electrolyte solvent. After 50 cycles, a substantial increase in the relative intensities of the C─O and O═C─O peaks was observed on the SiGr electrode in the case of the non‐fluorinated electrolyte. This observation strongly suggests an increasing amount of organic carbonate decomposition products during galvanostatic cycling. The relative intensity of the C─C peak decreases, while the relative intensities of C─C/C═C and CO_3_ peaks stay approximately the same after 50 cycles. In the case of the cells with the fluorinated solvents after 50 cycles, a doubling of the relative C─O peak intensity is observed, while the overall O═C─O, CO_3,_ and CF_3_ peak ratios remain nearly constant. Notably, the relative C─C/C═C peak intensity decreased by roughly half. These observations indicate that the compositions of the initially formed SEIs differ considerably for both considered electrolytes, resulting in the poor electrochemical performance of cells containing the non‐fluorinated electrolyte.

In contrast and despite the observed differences in impedance/resistance, the C 1s spectra of LNMO electrodes harvested from the cells with the non‐fluorinated and fluorinated electrolytes are practically identical after 3 and 50 cycles as well as compared to each other, as shown in Figure S5 (Supporting Information). This observation suggests the formation of a thin CEI layer and that the non‐fluorinated and fluorinated solvents only have a minor effect on the CEI composition. Otherwise, a notable alteration of the intensity of the decomposition products such as C─O and O═C─O, would be expected during galvasnotatic cycling between the cells with different electrolytes and cycle numbers and a change of the PVDF peak regarding the thickness of the CEI. This observation is in line with the consistent M─O intensity observed in the O 1s spectra (Figure S6, Supporting Information). These findings indicate that in this case, the CEI composition is not the decisive factor for the differences in impedance properties observed on aged electrodes.

**Figure 9:**
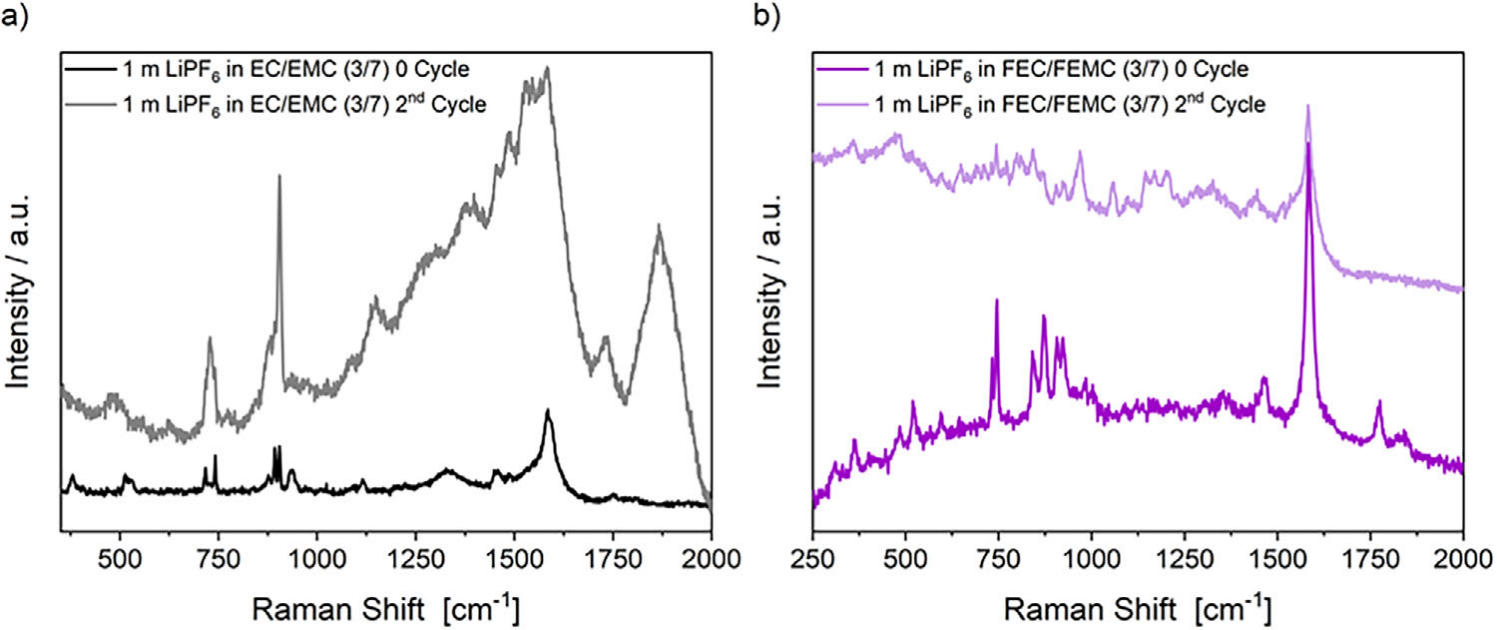
SHINER spectra recorded from the surface of a SiGr electrode in the presence of the 1 m LiPF_6_ in EC/EMC (3/7) a) and 1 m LiPF_6_ in FEC/FEMC (3/7) b) electrolyte. Spectra were recorded prior to galvanostatic cycling and after the second cycle.

*In situ* shell‐isolated nanoparticle‐enhanced Raman spectroscopy (SHINERS) measurements of the SiGr electrode surface were performed to obtain additional information on the influence of the non‐fluorinated and fluorinated electrolyte on interphase formation and to further elucidate the mechanisms of the SEI formation. Measurements were carried out in an optical cell with a 3‐electrode cell setup with SiGr as the working electrode, LNMO as the counter electrode and Li metal as the reference electrode directly after cell assembly (noted as 0 cycle) and then after the second cycle. The cells were charged/discharged with a sweep rate of 150 µV s^−1^ between 0.01 and 1.5 V versus Li|Li^+^. A comparison of the SHINER spectra of the cell with the non‐fluorinated and fluorinated electrolyte is shown in **Figure** **9**. The spectrum of the surface of the SiGr electrode with the non‐fluorinated electrolyte shows three prominent bands at ≈624, 1148, and 1378 cm^−1^ which can be assigned to semi‐carbonate‐like structures (ROCO_2_Li)^[^ ^43^ ^]^ and another distinct band at ≈1545 cm^−1^ which can be related to the carboxylate species.^[^ ^44^ ^]^ The laser decomposition product Li_2_C_2_ of organo‐Li species can be seen at ≈1866 cm^−1^.^[^ ^45^, ^46^ ^]^ The cell with the fluorinated electrolyte shows similar SEI components as ROCO_2_Li‐type species at ≈695, 800_,_ 1056_,_ 1166_,_ 1268_,_ 1437 and 1514 cm^−1^.^[^ ^47^, ^48^, ^49^, ^50^ ^]^ The bands in the areas between 500 and 800 cm^−1^ and 1100 and 1400 cm^−1^ can also be attributed to C─F vibrations, which could be an indication of fluorinated semi‐carbonates originating from the fluorinated electrolyte, which is in line with the XPS results.^[^ ^51^ ^]^ In addition, two bands can be observed at ≈684 and 969 cm^−1^ that indicate the presence of LiF,^[^ ^55^ ^]^ which was also detected in the XPS. Overall, the interphase formed with the fluorinated electrolyte shows some inorganic species, while the spectra recorded in the presence of the non‐fluorinated electrolyte show mainly the presence of organic species.

**Figure 10:**
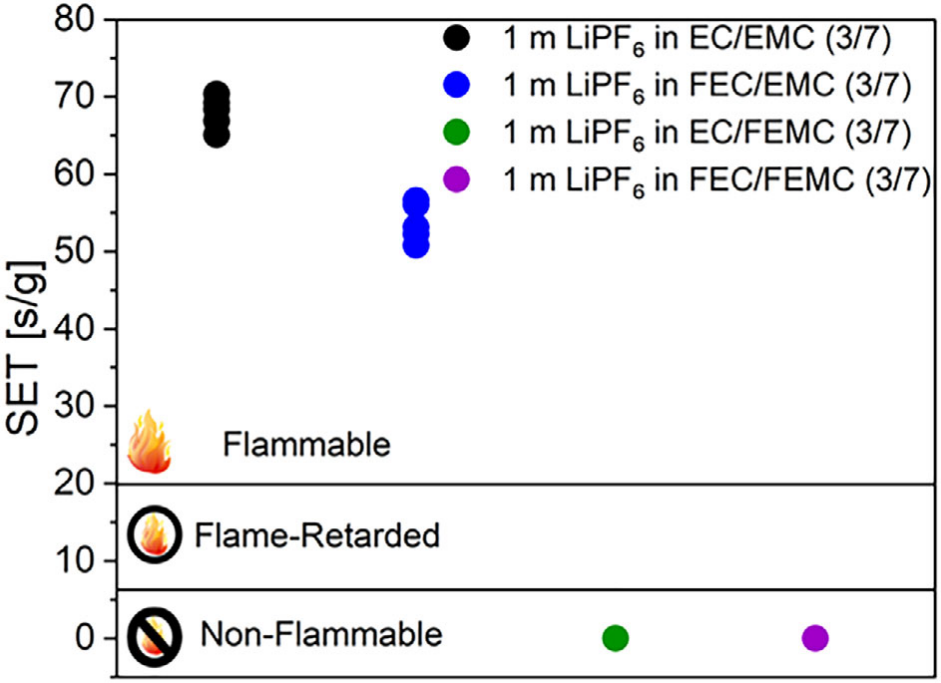
SET values  of four considered electrolyte formulations.

In addition to electrochemical performance, the presence of fluorine can considerably improve the safety properties of solvents for example by reducing their flammability.^[^ ^52^ ^]^ To evaluate the flammability of the four considered electrolyte formulations, self‐extinguishing time (SET) values was determined, as shown in **Figure** **10**. The SET of an electrolyte quantifies how long the electrolyte burns after the removal of the ignition source. Based on the SET values, electrolytes are classified into three categories: non‐flammable when SET <6 s g^−1^, flammable when SET >20 s g^−1^, or flame retarded if the SET value falls between 6 and 20 s g^−1^.^[^ ^53^ ^]^ The non‐fluorinated electrolyte exhibited a pronounced flammability with an SET of ≈68 s g^−1^. Substituting EC with FEC resulted in a lower burning duration, although the electrolyte remained flammable with an SET of ≈54 s g^−1^. The substitution of EMC by FEMC, resulting in EC/FEMC and FEC/FEMC formulations, effectively eliminated flammability, as evidenced by a SET value of 0 s g^−1^. The non‐flammability of these electrolytes can be attributed to the higher bond strength of C─F (109 kcal mol^−1^) compared to C─H (98 kcal mol^−1^) leads to a higher thermal stability of the fluorinated solvent.^[^ ^12^, ^54^, ^55^ ^]^ Additionally, replacing H atoms with F atoms in the solvent molecules, reduces the formation of H· radicals, which play a key role in the combustion reaction.^[^ ^12^ ^]^ During combustion, the FEMC molecule generates fluorine radicals (F·) that can capture reactive radicals like H· or OH· and contribute to the suppression of the combustion chain reaction.^[^ ^54^, ^56^ ^]^

**Figure 11:**
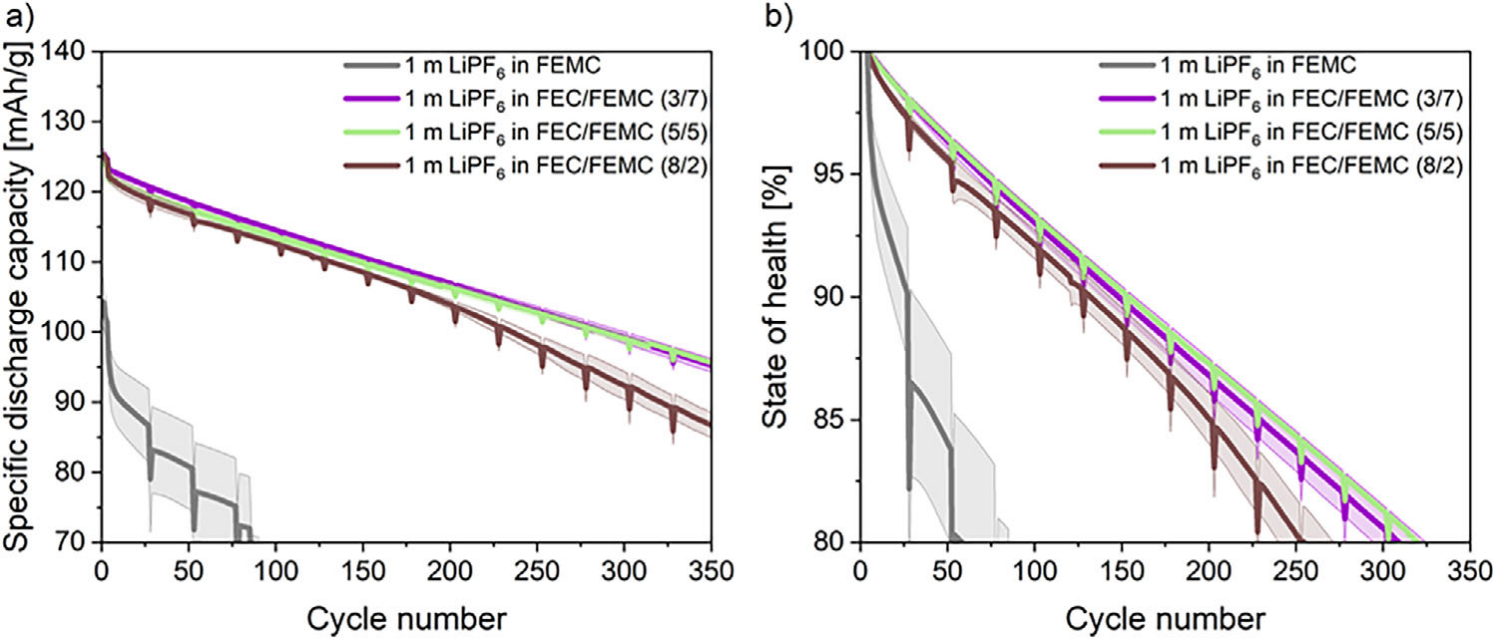
Specific discharge capacity a) and state of health b) of LNMO||SiGr (20 wt.% Si) cells as a function of the cycle number with different ratios of FEC and FEMC.

Due to environmental and health concerns regarding PFAS, the FEMC content in the electrolyte was systematically lowered to find the optimum balance between electrochemical performance and CF_3_ content.^[^ ^57^ ^]^ Seven electrolytes with 1 m LiPF_6_ as conducting salt and different amounts of FEC and FEMC as solvent/co‐solvent were formulated and the number of cycles until 80% SOH (**Figure** **11**) as well as the CF_3_ amount in the electrolyte formulation were calculated as shown in Table S1 (Supporting Information). A considerable reduction of the FEMC content to 1 m LiPF_6_ in FEC/FEMC (5/5) resulted in no noticeable decrease in electrochemical performance compared to the original electrolyte formulation while the CF_3_ content in the electrolyte was reduced from 21.9 to 15.6 wt.%. Further increase of the FEC/FEMC ratio resulted in a decline in electrochemical performance. Specifically, 1 m LiPF_6_ in FEC/FEMC (8/2), exhibiting a CF_3_ content of 6.3 wt.%, shows a limited cycle life of 256 cycles at 80% SOH compared to 310 cycles for the original formulation. The galvanostatic cycling data and the ionic conductivity data for other ratios of FEC/FEMC are shown in Figure S7 (Supporting Information). Electrolytes with a lower amount of FEMC have a specific discharge capacity of less than 100 mAh g^−1^ while cells using electrolytes containing only FEMC as a solvent can be galvanostatically cycled, but only achieve 50 cycles until 80% SOH. Consequently, it is necessary to use a mixture of FEC and FEMC to achieve optimum performance. However, it is noteworthy that all considered electrolyte formulations containing FEC and FEMC are non‐flammable. In summary, the optimal electrolyte formulation in terms of electrochemical performance, cost, and environmental aspects is 1 m LiPF_6_ in FEC/FECM (5/5).

Next to the flammability of the electrolyte, its toxicity is also essential with respect to safety. The electrolyte consisting of 1 m LiPF_6_ in EC/EMC (3/7) is known to inhibit acetylcholinesterase (AChE),^[^ ^58^ ^]^ an enzyme of the peripheral nervous system, influencing synaptic transmission. AChE cleaves the neurotransmitter acetylcholine (ACh) into acetate and choline in the synaptic cleft to prevent overstimulation, e.g., of the parasynaptic nervous system or the muscles. Symptoms of overstimulation can be a slow heart rate, paralysis, miosis, and respiratory failure, to name only a few.^[^ ^59^ ^]^ The conducting salt LiPF_6_ can react with the organic solvents to organo(fluoro)phosphates (OFPs), which have similar structural properties as chemical warfare agents and pesticides.^[^ ^60^ ^]^ These OFPs bind covalently to the serine hydroxy group in the active site of AChE and thus hinder the binding and cleavage of ACh.^[^ ^61^ ^]^ Different additives such as lithium difluorophosphate (LiDFP), FEC, and vinylene carbonate (VC) have been shown to modify the strength of the AChE‐inhibitory effects of 1 m LiPF_6_ in EC/EMC (3/7).^[^ ^58^ ^]^ The use of FEC as a solvent instead of as a functional additive raises the question of whether the beneficial effects on AChE inhibition persist.

**Figure 12:**
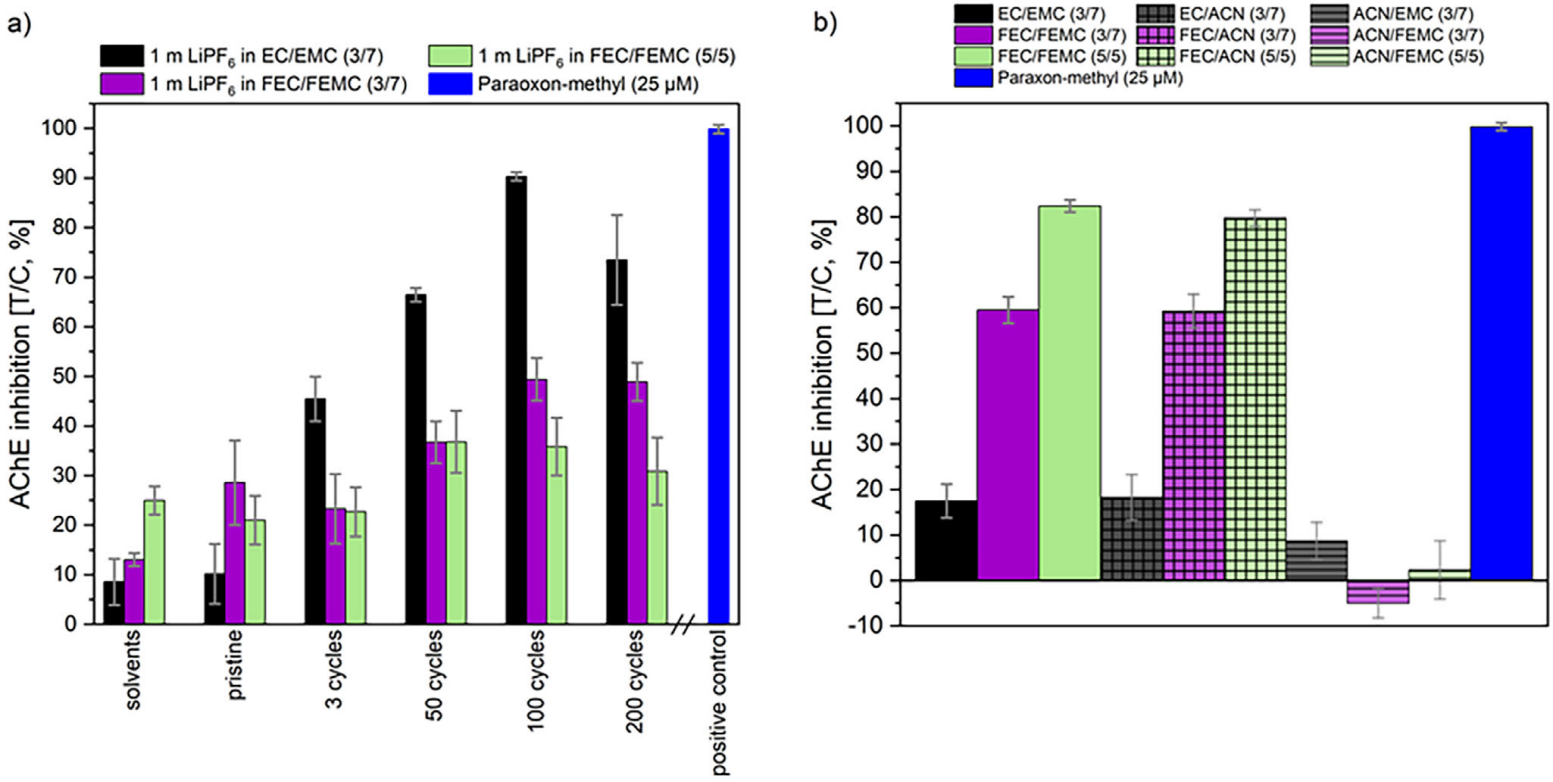
AChE inhibition [T/C, %] of 200 ppm solvent mixture or electrolyte after different numbers of cycles a) and AChE inhibition [T/C, %] of 2000 ppm of the solvent mixtures as applied in the electrolytes as well as the individual solvent/ACN mixtures b).Considered electrolytes: 1 m LiPF_6_ in EC/EMC (3/7), 1 m LiPF_6_ in FEC/FEMC (3/7), and 1 m LiPF_6_ in FEC/FEMC (5/5). Paraoxon‐methyl (25 µm) was used as a positive control.

The inhibition of AChE of 1 m LiPF_6_ EC/EMC (3/7), 1 m LiPF_6_ FEC/FEMC (3/7) and 1 m LiPF_6_ FEC/FEMC (5/5) was investigated at different ageing stages. An increasing trend can be observed for the AChE‐inhibitory potency of all electrolyte formulations with increased ageing (**Figure** **12**a). Nevertheless, the evaluated electrolytes show substantial differences. The most prominent inhibition was determined for the electrolyte based on EC/EMC (3/7), while FEC/FEMC (3/7) and FEC/FEMC (5/5) on the one hand started at higher inhibition rates for the pristine electrolyte but on the other hand, did not reach the effect level of EC/EMC (3/7) for any amount of ageing. To explain this behavior, two phenomena have to be considered. First, the higher inhibitory effects of the pure solvents and the pristine electrolyte for FEC/FEMC (3/7) and FEC/FEMC (5/5) suggest a stronger AChE inhibition by FEC in comparison to the other investigated solvents. This was confirmed in an additional experiment using the solvents without conducting salt and the individual solvent/ACN mixtures (Figure 12b). Only the mixtures containing FEC or EC were characterized as AChE inhibitors, of which FEC showed greater potency, while FEMC and EMC did not affect enzyme activity. These findings provide new mechanistic insights because the substances do not show any structural similarities to known AChE inhibitors.^[^ ^61^ ^]^ In the pristine electrolyte, the slightly higher inhibition in comparison to the pure solvents can be attributed to the presence of LiPF_6_ and the beginning formation of OFPs due to traces of moisture. In addition, higher levels of AChE inhibition can be observed for the electrochemically aged electrolytes than for the pristine electrolytes. The strong reduction of enzyme activity was ascribed to the formation of OFPs during galvanostatc cycling.^[^ ^58^, ^62^ ^]^ Up to 100 cycles, the results suggest an ongoing formation of OFPs, whereas their content appears to be stagnating afterward.

The differently aged electrolytes showed an opposite trend compared to the pristine formulations. EC/EMC (3/7) had a stronger effect than the fluorinated electrolytes, and of these, FEC/FEMC (3/7) showed a slightly higher inhibition than FEC/FEMC (5/5). An explanation of this behavior can be found in the nature of FEC, which forms longer and more stable polymers in comparison to EC. The resulting OFPs are polymerized to a higher degree hindering the binding into the active site of the enzyme. This effect has already been reported for FEC used as an additive in thermally aged electrolytes.^[^ ^58^ ^]^ A further explanation could be better incorporation of the OFPs with longer side chains into the interphase of the electrodes, leading to an overall lower amount of OFPs in the electrolyte.

In general, single FEC is a more efficient AChE inhibitor than EC, leading to a higher AChE‐inhibitory property of pristine fluorinated electrolytes. However, the results show a complete reversal of the effect for the harvested electrolytes, which highlights the importance of including toxicity investigation at different ageing stages of electrolytes for hazard characterization. Evaluation of raw materials is not sufficient as decomposition processes can considerably alter the toxicity. In summary, an enhanced FEC amount correlates with a lower impact of the harvested electrolyte on AChE‐activity and hence with a reduced toxicity.

## 3 Conclusion

The study focuses on evaluating the impact of FEC and FEMC as electrolyte solvents in combination with LiPF_6_ as conducting salt for LNMO||SiGr (20 wt.% Si) cell chemistry. With the combination of both fluorinated solvents in one formulation, an improvement in cycle life until 80% SOH of 56% was observed. Detailed electrochemical analysis demonstrated a prevented rollover effect and suppressed side reactions, resulting in an effective SEI and CEI formation, as confirmed by EIS measurements. Furthermore, XPS and SHINERS analysis of aged electrodes showed an increased amount of organic decomposition products on the SEI when using the non‐fluorinated electrolyte. This led to the formation of an ineffective SEI layer, which contributes to the observed poor electrochemical performance. Furthermore, the combination of FEC and FEMC enabled the realization of a non‐flammable electrolyte with decreased AChE inhibition by aged electrolytes compared to the non‐fluorinated counterpart. Given the concerns about PFAS such as CF_3_‐containing substances, it has been demonstrated that by fine‐tuning the electrolyte formulation it is possible to considerably reduce the CF_3_ content in the electrolyte while maintaining excellent electrochemical performance, non‐flammable nature of the electrolyte and even reducing toxicity.

## 4 Experimental Section

### Electrolyte Components and Resulting Formulations

The electrolyte components ethyl methyl carbonate (EMC), ethylene carbonate (EC), fluoroethylene carbonate (FEC), and lithium hexafluorophosphate (LiPF_6_) were purchased in battery grade quality from E‐Lyte innovation GmbH. Methyl 2,2,2‐trifluorethyl carbonate (FEMC) was purchased from E‐Novation Chemicals LLC with a purity of >99% and was purified by drying over CaH_2_ followed by distillation.

The electrolytes were formulated per molality (mol/kg) in an argon‐filled glovebox (MBraun, H_2_O, and O_2_ content below <1 ppm). 1 m LiPF_6_ in EC/EMC (3/7) was used as a reference electrolyte.

### Cell Assembly

Commercially available LNMO||SiGr (20% Si) dry two‐electrode pouch cells with a nominal capacity of 250 mAh from Li‐FUN Technology were used for galvanostatic cycling measurements. The cells were first cut open and dried for 14 h at 120 °C under reduced pressure. Thereafter, 700 µL electrolyte was added and the cells were sealed for 5 s under reduced pressure with a pouch cell sealer (GN‐HS200V) in a dry room. (dew point <−50 °C). A specially developed holder was used for the cells and a constant pressure of ≈2 bar was applied using a torque screwdriver. Three cells were assembled for each electrolyte formulation for reproducibility validation.

Swagelok T‐cells were assembled in a 3‐electrode configuration^[^ ^63^ ^]^ in the glovebox. The cell assembly comprised a 12 mm diameter working electrode, a Whatman GF/D glass fiber separator (ø 13 mm and ø 10 mm), lithium metal as reference electrode (ø 8 mm), and 200 µL electrolyte.

Symmetric CR2032 coin‐cells with two electrodes were assembled in a glovebox with 40 µL of electrolyte. The assembly followed this procedure: negative case, wave spring, 1 mm spacer, electrode (ø 12 mm), 20 µL electrolyte, Celgard 2320 (ø 16 mm), 20 µL electrolyte, electrode (ø 12 mm), 0.5 mm spacer, positive case. The cells were sealed using an automatic coin cell crimper (Hohsen Corp.) applying 7 kN for 5 s.

### Electrochemical Performance Evaluation

Galvanostatic cycling experiments were performed on a battery tester (Maccor Series 4000) in a temperature‐controlled chamber at 20 °C. After a rest step of 6 h, three formation cycles at C/10 with constant current/constant voltage (CCCV) steps in a voltage range between 3.5 and 4.9 V were performed. During further galvanostatic cycling, 24 cycles were conducted at a rate of 0.5 C, utilizing a constant current‐constant voltage (CCCV) step. After every 24 cycles, a capacity check was performed at 1 C, also employing a CCCV step.

### Electrochemical Impedance Spectroscopy

Electrochemical impedance spectroscopy (EIS) measurements were performed on a Bio‐Logic VMP3 workstation. After a resting period of 30 min, the cell was measured in a frequency range from 1 mHz to 1 MHz. The Li‐FUN cells were galvanostatically cycled for 3 and 50 cycles and then charged to a 50% state of charge (SOC). Then the cells were disassembled, and the electrodes (ø 12 mm) and symmetric coin cells with 40 µL electrolyte of the same electrolyte were assembled.^[^ ^32^, ^36^ ^]^

### Laser Ablation‐Inductively Coupled Plasma‐Mass Spectrometry

To investigate transition metal dissolution, the anodes were measured using a laser ablation‐inductive coupled plasma‐mass spectrometer. LA‐ICP‐MS imaging measurements were performed using a 193 nm ArF excimer laser (Analyte Excite Excimer LA‐System, Teledyne CETAC, Omaha, NE, USA) coupled to an ICP‐MS system (7700 Series, Agilent Technologies, Santa Clara, CA, USA) via an Aerosol Rapid Introduction System (ARIS, Teledyne CETAC Technologies). The laser was operated at a frequency of 50 Hz with a spot size of 50 µm, 12.5x scan speed (625 µm s^−1^), and laser fluence of 3 J cm^−2^. The ICP was used with an RF Power of 1550 W, 1.6 V RF Matching, a carrier gas flow of 1.0 L min^−1^, and without an optional gas flow. In order to correct varying ablation rates, transport efficiencies, and different plasma conditions, the respective TM signal was divided by the ^13^C signal, since carbon was homogenously distributed in the anode.

### Ionic Conductivity Determination

For ionic conductivity experiments, 2 mL Eppendorf Safe‐Lock tubes were filled with 750 µL of electrolyte. EIS measurements were performed with custom‐designed electrodes as described in our previous work,^[^ ^64^, ^65^ ^]^ connected to a Metrohm Autolab/M204 potentiostat/galvanostat. Samples were equilibrated in a temperature chamber (Memmet TTC256, with a temperature setting accuracy of 0.1 °C) for 2 h at each target temperature. Measurements were performed over a temperature range of – 30 °C to 60 °C in 10 °C intervals. The ionic conductivity of the electrolyte was determined by fitting impedance spectra using Metrohm Nova software with a model that has set parameters for the resistors *R_S_* and *R_P_*, as well as for the constant phase element (CPE). And the fitting model *R_S_* (CPE‐*R_P_*) was used. The fitting was performed after each data point, and the electrolyte conductivity was calculated from the quotient of the cell constant and the determined electrolyte resistance.^[^ ^66^ ^]^

### Self‐Extinguishing Time Determination

For the self‐extinguishing time (SET) determination experiments, 4 Whatman GFD separators (ø 12 mm) were placed on a cannula which was attached to the laboratory stand with a clamp. The separator was wetted with 400 µL electrolyte and thereafter lit with a lighter for 10 s. The distance between the lighter and the separator was 3.5 cm. The time until the flame was extinguished was measured.

### X‐ray Photoelectron Spectroscopy

XPS measurements were carried out with a photoelectron spectrometer (K‐Alpha, Thermo VG Scientific). Monochromatic Al‐Kα X‐rays were used for the measurements (Al Kα, hν  =  1486.6 eV). The base pressure of the measuring chamber was typically ≈2⋅10^−9^ mbar. The device was regularly calibrated with regard to the energy position in accordance with ISO 15 472 using copper, silver, and gold reference samples. The intensity scale was calibrated according to the device manufacturer's specifications. All spectra were decomposed using a pseudo‐Voigt function with 70% of Gaussian and 30% of Lorentzian distribution. Binding energies were calibrated using the C 1s peak position corresponding to graphitic carbon at 284.5 eV.

### Shell‐Isolated Nanoparticle‐Enhanced Raman Spectroscopy

The nanoparticles used for the presented shell‐isolated nanoparticle‐enhanced Raman spectroscopy (SHINERS) analysis were prepared as described in the studies by *Pfeiffer* et al.^[^ ^35^ ^]^ For the transfer of the NPs to the electrode surface, the NPs were transferred from the aqueous synthesis medium to an organic medium (isopropanol). After transfer, the NPs were drop cast onto SiGr electrodes and harvested from LNMO||SiGr Li‐FUN pouch cells. For this, the Li‐FUN cell was opened, and one side of the double‐side coated SiGr was removed with water, then punched into 12 mm electrodes and dried for 12 h at 120 °C under reduced pressure. In total 250 µL of concentrated NP solution were added to the electrode. Afterward, the electrodes were dried under a reduced atmosphere for 12 h at 90 °C and transferred to an argon‐filled glovebox (MBraun, O_2,_ and H_2_O content <5 ppm) for storage.

SHINERS measurements were performed using ECC‐Opto‐STD optical cells from EL‐CELL. As a working electrode, the SiGr electrode with NPs was connected at the bottom of the cell facing toward the optical window. A separator (Celgard 2500, PP) with a circular cut‐out in the center was placed on top of the electrode to allow observation of the SiGr electrode surface. As a counter electrode, a half‐circle‐shaped LNMO electrode was placed above, facing the bottom of the cell with its active material. Note that the LNMO electrode did not cover the cut‐out within the separator. Li‐metal was introduced as a reference electrode on the side. After electrode stacking, the optical cell was filled with ≈200 µL of the respective electrolyte.

Potentiostatic cycling of the optical cells was performed using an Autolab potentiostat/galvanostat (Metrohm) controlled by the Nova 2.1 software (Metrohm). The cells were charge/discharge with a scan rate of 150 µV s^−1^ between 0.01 and 1.5 V versus Li|Li^+^.

Raman measurements were performed before the first and after the second charge/discharge cycle, using a confocal Raman microscope (LabRam HR evolution, Horiba Scientific) equipped with an air‐cooled CCD detector and 600 line mm^−1^ grating. The samples were excited using a 633 nm laser with a power of 1.05 mW. Spectra were acquired 4 times over 25 s. Handling of the Raman spectrometer and curation of the obtained spectra was performed using the LabSpec 6.7.2.1 software (Horiba Scientific). Prior to the Raman experiments, the system was calibrated onto the band of c‐Si at 520.70 cm^−1^.

### Toxicological Assessment

Mono‐ and dibasic potassium phosphate, acetylcholinesterase lyophilizate from *Electrophorus electricus* (electric eel) (AChE), 5,5′‐dithiobis(2‐nitrobenzoic acid) (DTNB), acetylthiocholine iodide (ATCh) and acetonitrile (ACN) were purchased from Sigma–Aldrich.

Evaluated formulations and solvent mixtures, namely 1 m LiPF_6_ in EC/EMC (3/7) and 1 m LiPF_6_ in FEC/FEMC (3/7) as well as 1 m LiPF_6_ in FEC/FEMC (5/5) each in six different (electrochemical) ageing stages as shown in **Table** **1**. In the second setup, the mixed solvents without conducting salt were compared to the individual solvents mixed with ACN in the respective ratios (3/7, 5/5) in order to determine their individual AChE inhibiting capability.

**Table 1:** Overview of the considered compounds of the pristine electrolyte and the aged electrolyte after different numbers of cycles.

Solvent mixture/electrolyte formulation	Pristine electrolyte	Aged electrolyte after different number of cycles:			
		3	50	100	200
EC/EMC (3/7)	X				
FEC/FEMC (3/7)	X				
FEC/FEMC (5/5)	X				
1 m LiPF_6_ in EC/EMC (3/7)	X	X	X	X	X
1 m LiPF_6_ in FEC/FEMC (3/7)	X	X	X	X	X
1 m LiPF_6_ in FEC/FEMC (5/5)	X	X	X	X	X

Inhibition toward *Electrophorus electricus* (electric eel) AChE was evaluated using the *Ellman Assay* ^[^ ^67^, ^68^ ^]^ as described by *Kubot* et al.^[^ ^58^ ^]^ with slight modifications. The assay was conducted in 96‐well plates and the reagents were diluted to their final concentrations in potassium phosphate (PP) buffer (pH 8).

The electrolytes or their individual components were diluted in a mixture of ACN and PP buffer in order to reach final concentrations of 200 ppm electrolyte in the assay, or 2000 ppm solvent/ACN mixture. ACN concentration was ≈2% in each single experiment. ACN was used as negative/blind and paraoxon‐methyl (25 µm) as positive control, respectively. For starting conditions (t  =  0 min) 50 µL of the sample/ACN/PP‐mix and 50 µL AChE solution (0.2 units mL^−1^) or PP buffer in case of the blind control, were transferred to a 96‐well plate. The plate was shaken on a laboratory shaker (5 min, 300 U min^−1^). At t  =  8.5 min, 20 µL of the freshly mixed DTNB (1 mmol L^−1^)/ATCh (1 mmol L^−1^) solution was added to each well. Afterward, the plate was shaken again for 1 min. At t  =  17 min, absorbance was measured using the Tecan Infinite 200 PRO microplate reader at 412 nm and 25 °C (software: Tecan i‐control V1.7 SP1). The absorbance of each well was corrected by the average of the blind measurements, and the results were calculated as AChE inhibition [T/C, %]. Experiments were conducted in triplicates.

## Conflict of Interest

The authors declare no conflict of interest.

## Supporting information



Supporting Information

## Data Availability

The data that support the findings of this study are available from the corresponding author upon reasonable request.
