# Supplementary material for: Importance of Fluorine in High Voltage Electrolytes for LNMO||SiGr Cell Chemistry
Source: Small. 2025 Jul 10;21(35):2505254. doi: 10.1002/smll.202505254 (PMC12410896; doi:10.1002/smll.202505254)

**Importance of fluorine in high voltage electrolytes for LNMO||SiGr cell chemistry**

Maike Leopold^1^, Felix Pfeiffer^1^, Elisabeth Christine Muschiol^2^, Christian Wölke^1^, Peng Yan^1^, Kai Brüning^3^, Sascha Nowak^3^, Melanie Esselen^2^, Martin Winter^1,3^, Isidora Cekic-Laskovic^1*^

1: Helmholtz-Institute Münster (IMD-4), Forschungszentrum Jülich GmbH, Corrensstraße 48, 48149 Münster, Germany

2: Institute of Food Chemistry, University of Münster, Corrensstraße 45, 48149 Münster, Germany

3: MEET Battery Research Center, University of Münster, Corrensstraße 46, 48149 Münster, Germany

**Supporting Information**

**
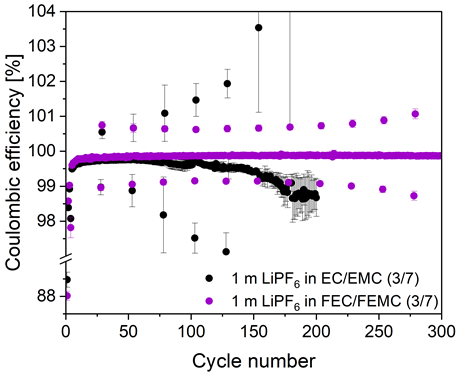
**

**Figure S1:** Coulombic efficiency as a function of cycle number of the LNMO||SiGr cells containing non-fluorinated and fluorinated electrolytes.


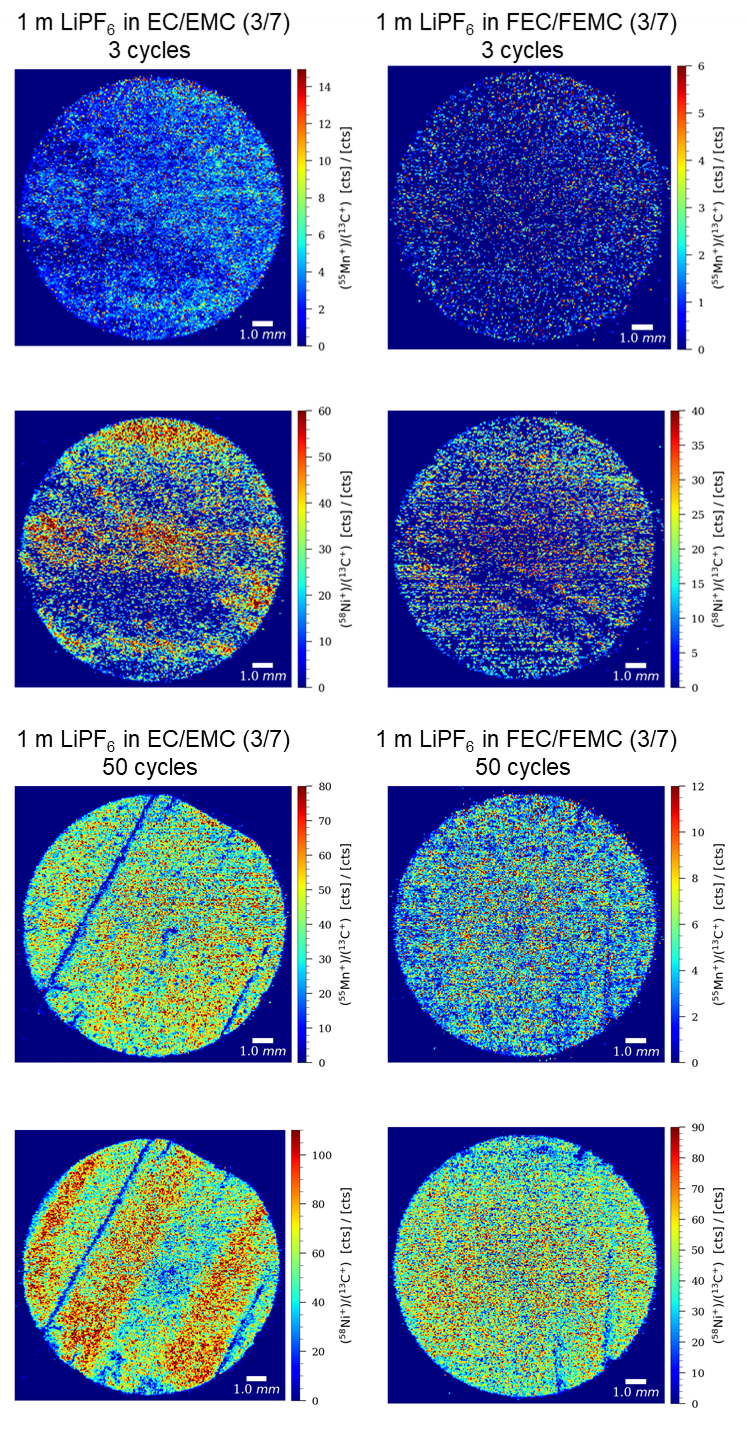


**Figure S2:** LA-ICP-MS element mapping for Mn and Ni on SiGr anode surface harvested from LNMO||SiGr cells with non-fluorinated and fluorinated electrolytes after 3 and 50 cycles.


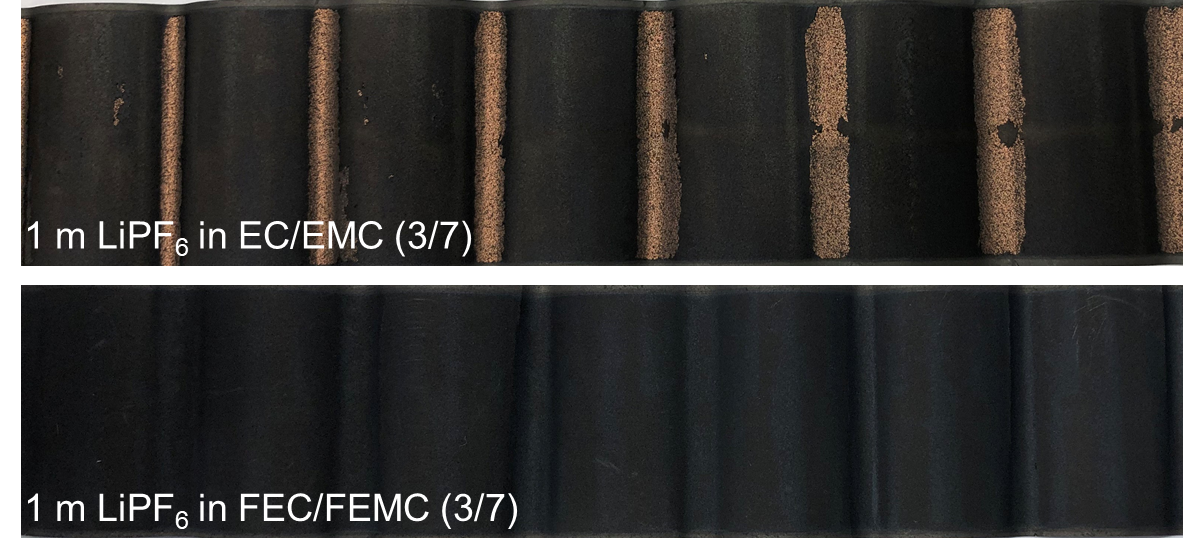


**Figure S3**: Photographs of the SiGr electrode harvested from the LNMO||SiGr Li-FUN cell with 1 m LiPF_6_ in EC/EMC (3/7) (a) and 1 m LiPF_6_ in FEC/FEMC (3/7) electrolytes (b) after 50 cycles.


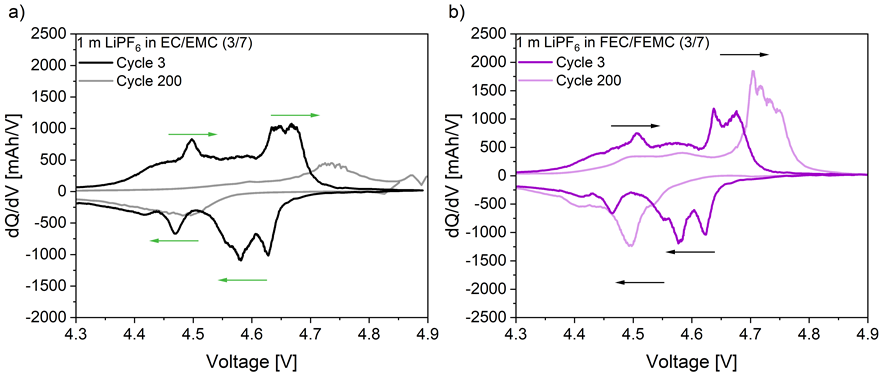


**Figure S4:** Differential capacity versus cell voltage plots of LNMO||SiGr (20 wt.%) cells containing (a) non-fluorinated solvents and (b) fluorinated solvents at 4 different cycles.


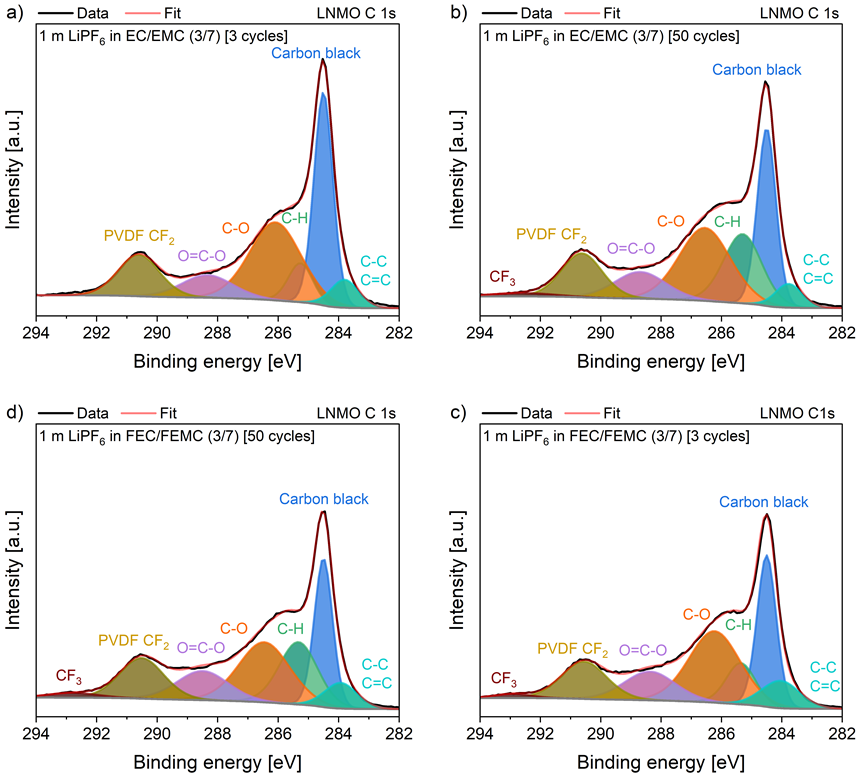


**Figure S5:** Fitted core C 1s spectra for harvested LNMO electrodes with non-fluorinated (a, b) and fluorinated electrolyte (c, d) after 3 and 50 cycles.


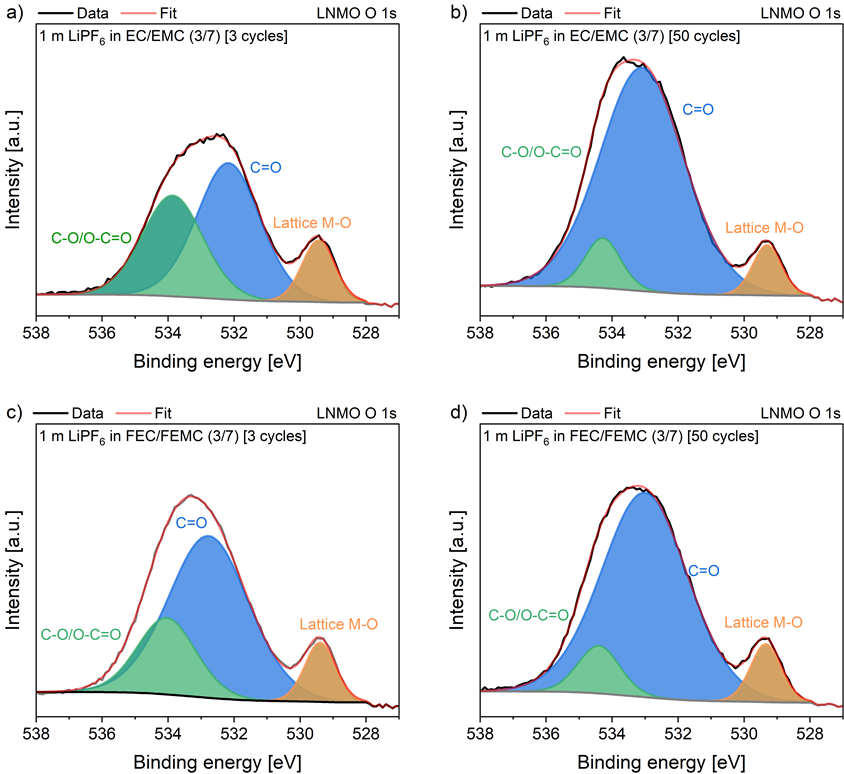


**Figure S6:** Fitted core O 1s spectra for harvested LNMO electrodes with non-fluorinated (a, b) and fluorinated electrolyte (c, d) after 3 and 50 cycles.


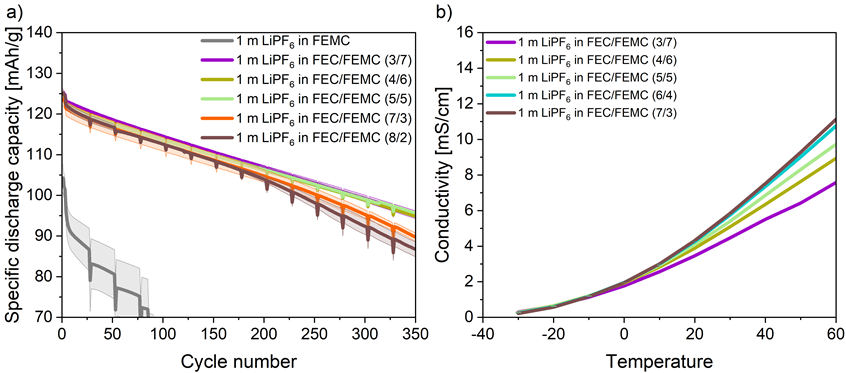


**Figure S7:** Specific discharge capacity as a function of cycle number (a) and ionic conductivity as a function of the different temperatures from -30 °C to 60 °C (b) with the different ratios of FEC to FEMC.

**Table S1:** CF_3_ content and number of cycles at 80 % SOH seven electrolyte formulations with different amounts of FEC and FEMC.


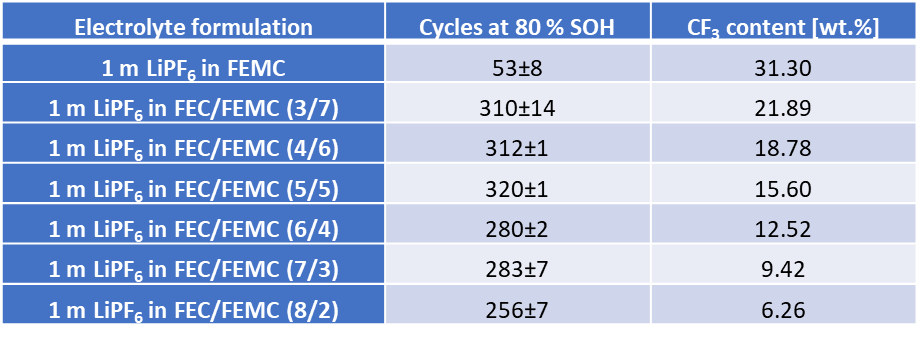

Supplement: Supplementary file 1 — Supporting Information [file SMLL-21-2505254-s001.docx]
